# Critical Role of Diels–Adler Adducts to Realise Stretchable Transparent Electrodes Based on Silver Nanowires and Silicone Elastomer

**DOI:** 10.1038/srep25358

**Published:** 2016-05-03

**Authors:** Gaeun Heo, Kyoung-hee Pyo, Da Hee Lee, Youngmin Kim, Jong-Woong Kim

**Affiliations:** 1 Display Materials & Components Research Center, Korea Electronics Technology Institute, 68 Yatap-dong, Bundang-gu, Seongnam 463-816, South Korea

## Abstract

This paper presents the successful fabrication of a transparent electrode comprising a sandwich structure of silicone/Ag nanowires (AgNWs)/silicone equipped with Diels–Alder (DA) adducts as crosslinkers to realise highly stable stretchability. Because of the reversible DA reaction, the crosslinked silicone successfully bonds with the silicone overcoat, which should completely seal the electrode. Thus, any surrounding liquid cannot leak through the interfaces among the constituents. Furthermore, the nanowires are protected by the silicone cover when they are stressed by mechanical loads such as bending, folding, and stretching. After delicate optimisation of the layered silicone/AgNW/silicone sandwich structure, a stretchable transparent electrode which can withstand 1000 cycles of 50% stretching–releasing with an exceptionally high stability and reversibility was fabricated. This structure can be used as a transparent strain sensor; it possesses a strong piezoresistivity with a gauge factor greater than 11.

An electrode based on a networked structure of randomly distributed silver nanowires (AgNWs) and a stretchable polymer is known to have a high potential for use in various mechanical sensing applications such as strain gauges for robotics, force sensors, human motion detectors, and stretchable touch sensors. Highly dense nanowires embedded in the surface of a stretchable polymer can reportedly result in a mechanically robust electrode which can withstand large strains of up to 30–80% without losing conductivity^1^,^2^,^3^,^4^,^5^. This may be due to the undamaged nanowires cancelling the stretching effect on damaged wires at high densities. However, there is a strong need to develop a stretchable electrode containing sparsely dispersed nanowires from two important perspectives: the transparency of the electrode and the gauge factor (GF). The transparency of an electrode can generally be enhanced by lowering the nanowire density, and the resulting electrode can be employed during fabrication in a wide variety of applications^6^,^7^,^8^,^9^. More importantly, the GF has been reported to be higher when the AgNW density in the electrode is decreased^10^. This may also be related to the cancellation effect in that the electrically disconnected AgNWs are offset by the reconnection of nanowire bundles. This implies that a mechanically robust stretchable electrode with both a high transparency and a high GF needs to be developed to realise a reliable transparent strain sensor.

Silicones have received much attention as polymers for oils, coating materials, and films because of their flexibility, hydrophobicity, and heat resistance. The combination of polydimethylsiloxanes (PDMSs) with functional groups is a versatile method for producing various silicone-based polymers. For instance, Sylgard 184 consisting of silane and vinyl silane was cured in the presence of a Pt catalyst to yield the silicone 184 PDMS^11^. Even though this is stretchable and transparent, the low surface energy discourages its use as a substrate for stretchable electrodes. For instance, when an AgNW network is formed on 184 PDMS, it is easily peeled from the polymer by gentle rubbing or a moderate mechanical stress. To incorporate AgNWs onto the silicone film, the surface of 184 PDMS has been treated with primers or an oxygen plasma to enhance its wetting properties. Although the obtained electrode for which the conductors can interact with primers via hydrogen bonding displayed decent electrical properties, its physical properties were dependent on the thickness and properties of the primer layer. Therefore, employing an identical material (184 PDMS) as a cover or protective layer may help in achieving a more reliable and stretchable transparent electrode based on AgNWs and a polymer.

A multi-layered 184 PDMS film is difficult to fabricate because of the lack of anchoring sites among silicone layers. Some groups have reported that curing a 184 PDMS resin on a partially cured 184 PDMS layer leads to a multi-layered structure^10^,^12^,^13^. The remaining curing sites of the soft-cured 184 PDMS participate in the curing reaction of the resin overcoat. Here, we report the use of Diels–Alder (DA) adducts as crosslinkers to fabricate a silicone/AgNW/silicone structure. A silicone polymer featuring furans was synthesised and crosslinked by making use of maleimides to produce PU-1A. The cyclisation reaction between furan and maleimide leads to the formation of DA adducts. Because the DA reaction is reversible, the unreacted furans and maleimides should remain after the reaction. Therefore, the crosslinked film (PU-1A) can participate in the DA reaction with the resin overcoat to bond with the polymer. Thanks to a water contact angle of 87° (Supplementary Figure S1), AgNWs dispersed in isopropyl alcohol (IPA) can be coated onto a layer of PU-1A. In addition, the hydrogen donor of the urethane (NHCOO) unit of PDMS-based polyurethane can interact with AgNWs to improve the mechanical stability even when the electrode is stretched multiple times^14^. One more polymer coating onto AgNW/PU-1A yields the PU-1A/AgNW/PU-1A structure, which should seal the electrodes so that any surrounding liquid cannot leak through the interfaces. Furthermore, the nanowires can be protected by the PU-1A overcoat when they are stressed by various mechanical loads such as bending, folding, or stretching. On this basis, we were able to fabricate a stretchable transparent electrode which could withstand 1000 cycles of 50% stretching and releasing with exceptionally high stability and reversibility. Enabled by the complete sealing of the electrodes, a sparsely dispersed AgNWs in low density could be successfully integrated to achieve both high GF and transparency.

## Results and Discussion

**Figure 1:**
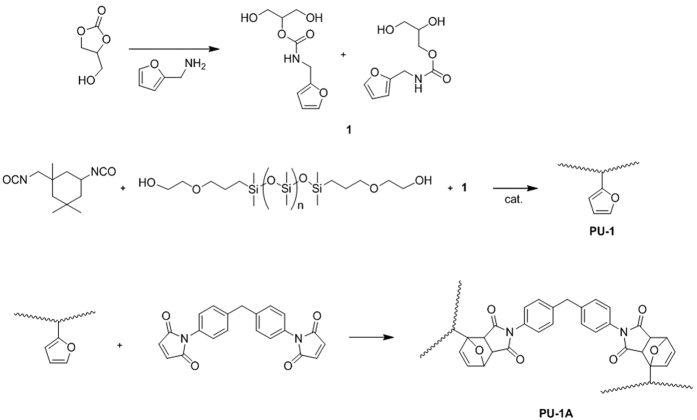
Synthesis of PU-1A.

Hydroxyl-terminated silicone polyol was employed to synthesise the silicone-based polymer (Fig. 1). The reaction between isophorone diisocyanate (IPDI) as the isocyanate and alcohol at the NCO/OH reaction ratio of 1:1 yielded polyurethane (PU-1). The alcohol derivatives comprised **1**^15^ as a hard segment and the PDMS-based polyol as a soft segment at the OH/OH equivalent ratio of 5:5. The reduced intensity in the infrared (IR) absorption band at 2260 cm^−1^ corresponding to NCO stretching indicated that the reaction proceeded (Supplementary Figure S2). After the reaction was completed, the number average molecular weight of PU-1 was measured to be 3980 g mol^−1^ with PDI (M_w_/M_n_) = 23.29 by a gel permeation chromatography. Because of the lack of crosslinking, the polymer was easily soluble in organic solvents such as methyl ethyl ketone (MEK) and diethyl ether. The chemical and mechanical properties of PU-1 were improved through crosslinking. The furanyl groups of PU-1 and maleimide groups of 1,1′-(methylenedi-4,1-phenylene)bismaleimide (BMI) formed the DA adducts via [4 + 4] cycloaddition, which served as crosslinkers. The advantage of the furan/maleimide combination is the mild conditions for the DA and retro-DA reactions. Once BMI was completely dissolved in the solution of PU-1 in N,N-diemthylformamide (DMF), the mixture was coated onto glass and dried at 50 °C overnight to carry out the DA reaction. The crosslinked polyurethane (PU-1A) was insoluble in MEK and diethyl ether thanks to the dense crosslinking. Because of the poor solubility of the polymer, the presence of the DA adducts was confirmed after the sample was heated in N,N-dimethylformamide-d7 (DMF-d_7_) at 120 °C for 15 min (Supplementary Figure S3). Differential scanning calorimetry (DSC) was employed to investigate the thermal properties of PU-1A by increasing the temperature from −100 °C to 200 °C (Supplementary Figure S4). During the first heating process, several endothermic and exothermic peaks were observed. The peak at 27.5 °C was considered to represent *T*_*g*_ of PU-1A. The peak at 65 °C was attributed to the DA reactions between free furans and maleimides^16^. The exothermic band at 140–180 °C was presumed to stem from maleimide polymerisation^16^,^17^. Considering that the retro-DA reaction occurs above 100 °C, the generation of free maleimides followed by polymerisation is plausible. The second heating of this sample displayed a different DSC spectrum from the previous one, as shown in Supplementary Figure S4. There were two endothermic peaks at 26 and 130 °C, even though the latter was rather ambiguous. The former peak indicated that the polymer backbone was stable up to 200 °C, showing little change in *T*_*g*_. The TGA data showed good agreement with the DSC data, which indicates that the temperature for a 5% mass change is higher than 200 °C (Supplementary Figure S5).

**Figure 2:**
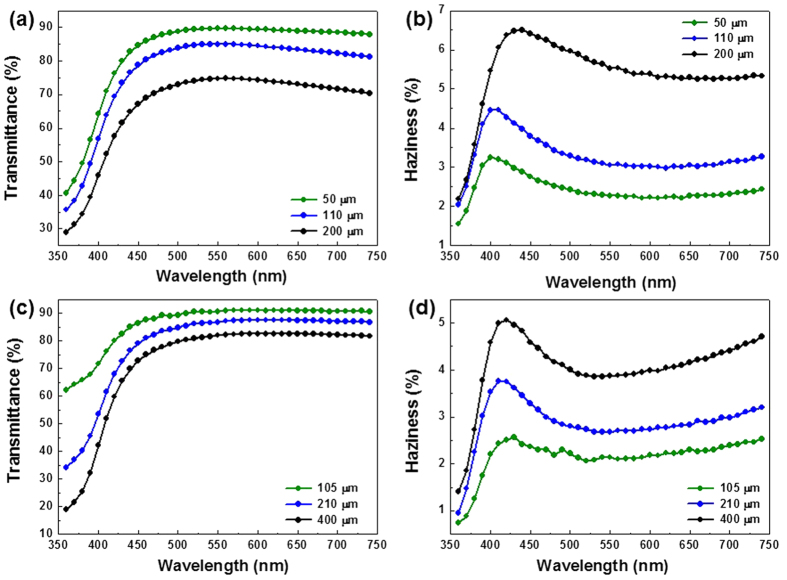
Optical properties of the electrodes. (**a,b**) Transmittance and haziness of AgNW/PU-1A (monolayer) and (**c,d**) transmittance and haziness of PU-1A/AgNW/PU-1A (sandwich). The legends in the graphs indicate the total thickness of the electrodes including the polymer and AgNWs.

By varying the spin-coating speed for PU-1, we fabricated PU-1A with three different thicknesses: 50, 110, and 200 μm. The 0.5 wt% AgNW dispersion was coated onto PU-1A preformed on glass and heated to 120 °C for 10 min. During the heating, the retro-DA reaction occurred to produce the free furans and maleimides. This resulted in a stretchable transparent electrode (monolayer). Figure 2a,b show the visible wavelength transmittance and haziness (i.e. the ratio of diffused transmission and total transmission), respectively, of the electrodes, including the transmittance of the polymer (the transmittance of the only AgNWs was also given in Supplementary Figure S6). Note that, even over a very broad spectral range of 450–700 nm, the film with the lowest thickness (50 μm) exhibited a high transmittance of more than 85%, and the sheet resistance *R*_*s*_ of the electrode (40 Ω/sq) was comparable to that of a AgNW network coated on glass. This performance is better than that of the commercially available ITO films (*R*_*s*_: 100 ohm/sq and transmittance: 88%) and comparable to those of AgNWs embedded at the surface of various polymers^8^,^9^,^14^,^15^. An increase in the thickness of PU-1A decreased the transmittance and increased the haziness of the electrode; this was mainly due to the greater light absorption and scattering. PU-1 was formed on the AgNW/PU-1A structure once more, which afforded the PU-1A/AgNWs/PU-1A structure after being kept at 50 ^o^C. When the polymer layer of PU-1A was formed via the DA reaction, the free furans and maleimides in PU-1A participated in the DA reaction. By this simple mechanism, the stacked PU-1A layers could be integrated to be a unity. Figure 2c,d show its transmittance and haziness, respectively. Interestingly, the PU-1A overcoat enhanced the optical performance of the electrodes: the transmittance of the sandwich sample with a thickness of 105 μm was higher than that of the monolayer sample with a thickness of 50 μm for wavelengths in the whole visible-light spectrum. The haziness was also remarkably decreased owing to the overcoat. This improvement has also been observed in the case of oxide/metal/oxide structures such as indium tin oxide (ITO)/Ag/ITO or TiO_2_/Ag/TiO_2_^18^,^19^,^20^,^21^. The transmission spectrum indicated that the dielectric/metal/dielectric structure is better than the metal/dielectric structure in terms of light transmission, which can be explained by the antireflection effect: the dielectric layer with the overcoat suppresses the reflection and scattering from the surface of the metal in the visible region. Analogising this to our system indicates that the PU-1A overcoat decreased the reflection and scattering of the visible light at the surface of the AgNWs; therefore, a higher transmittance and lower haziness were measured. In order to verify this, we also fabricated a sandwich structure of PU-1A/PU-1A without employing the AgNWs. The transmittance and haziness of this structure was compared with those of monolayer as shown in Supplementary Figure S7, in that the optical performances of the sandwich structure were not enhanced by the stacking, and this supports the hypothesis we discussed above.

**Figure 3:**
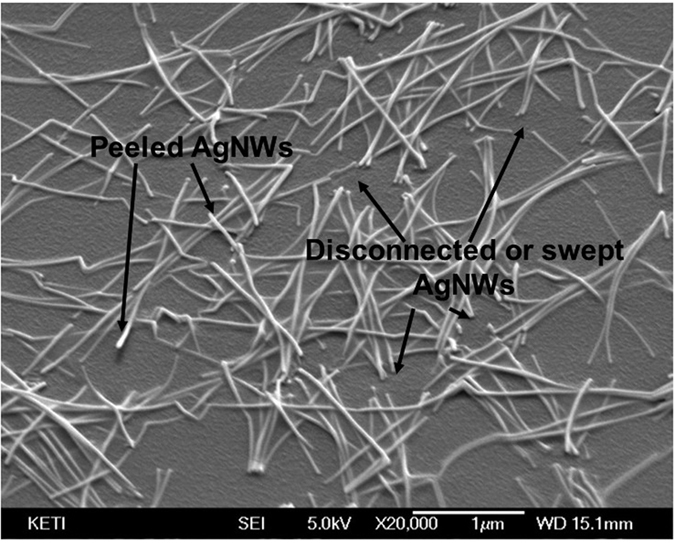
Failure modes of the AgNW/PU-1A electrode after the stretching test (a 30% strain was applied to the sample 10 times).

In order to evaluate the mechanical stability of the AgNWs coated on PU-1A during stretching, the samples were repeatedly stretched (strain: 30%) and released ten times and observed with a field-emission scanning electron microscope (FESEM). Figure 3 shows the various failure modes typically observed in this case (the original state of the electrode is also given in Supplementary Figure S8): delamination of AgNWs from the polymer surface and their disconnection. Because we did not employ any specific method to bury the nanowires into the surface of polymer, the AgNWs seemed to just be placed on the PU-1A and not properly adhered or embedded. In this case, the mechanical stability was not sufficient to resist stretching, and many broken nanowires are easily observed in Fig. 3. The instability of the percolated nanostructure originated from the insufficient adhesion between the constituents. For the AgNW/PU-1A structure, good adhesion and large contact areas between the AgNWs and PU-1A are the most important factors which prevented the nanowires from sliding at the interfaces^22^,^23^. This sliding is known to be the main cause of singularities at a stress distribution, which contributes to the disconnection of stripped nanowires^24^. In order to overcome this issue, various methods have been suggested to enhance the adhesion, such as an inverted layer process to fully bury the nanowire into the surface of polymer^25^,^26^,^27^,^28^, irradiation with high-energy pulsed light to selectively heat the nanowires and react with the underlying polymer^29^,^30^,^31^,^32^, and the use of an adhesive layer or binding materials^14^,^33^. The inverted layer process is known to be one of the most effective ways to enlarge contact areas and thus enhance the adhesion. However, this method needs to peel off and flip the fabricated electrode to deposit the overlying materials which provide a specific function or interconnection; this is very difficult to achieve without causing wrinkles or defects. Furthermore, the fully buried nanowires need to be excavated to enhance the connectivity with neighbouring layers, possibly through the use of a solution or dry-processed etching^28^.

**Figure 4:**
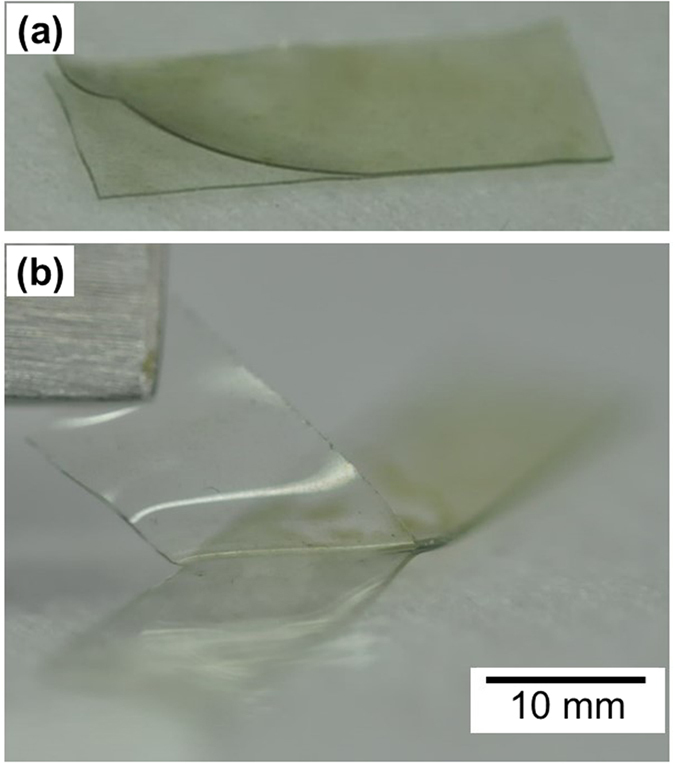
Sandwich sample comprising two layers of 184 PDMS: (**a**) after 1000 repeated stretches up to 30% strain and (**b**) easy peeling-off of the upper layer from the underlying polymer. Liquid 184 PDMS was coated onto another 184 PDMS layer partially cured at 70 °C for 20 min followed by full curing at 70 °C for 24 h.

**Figure 5:**
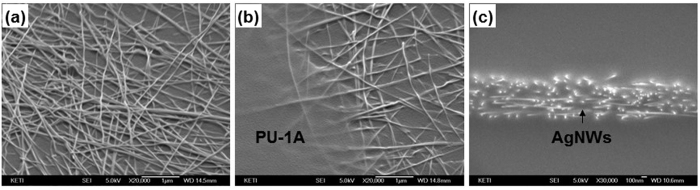
SEM images of the fabrication of the sandwich structure. (**a**) AgNW coating on cured PU-1A, (**b**) PU-1A partially covering AgNW/PU-1A, and (**c**) a cross-sectional view of the sandwich structure of PU-1A/AgNW/PU-1A.

The cover layer employed in this study is very useful for restraining the stress singularity which can form at the interfaces between the nanowires and the polymer when the electrode is mechanically strained, but only if the following two conditions are satisfied: (1) the two layers should be identical or at least have similar material properties and (2) the overlying polymer needs to strongly adhere to the underlying polymer. Because 184 PDMS fulfils these two prerequisites, it was used to fabricate the structure; an overcoat of liquid 184 PDMS was used to cover another 184 PDMS layer partially cured at 70 °C for 20 min. In order to integrate the two layers, the sample was fully cured at 70 °C for 24 h. The mechanical stability was tested by repeated stretching with a 30% strain for 1000 cycles. However, as shown in Fig. 4, the two layers were very easily separated by a mild peeling-off procedure (also shown in Supplementary Figure S9). This may have been due to the lack of remaining curing sites in the partially cured 184 PDMS. A similar structure was fabricated by using PU-1A according to the procedure described in Section 4. The AgNWs were deposited onto the cured PU-1A and dried as shown in Fig. 5a. In this sequence, the nanowires were loosely adhered, so they were easily detached by gentle rubbing. Several drops of PU-1 were cast onto AgNW/PU-1A, and the edge of the cured drops is shown in Fig. 5b. A closer look at the covered layer revealed a mild gradient of contrast for the wires, which implies that the cast PU-1 properly wetted AgNW/PU-1A. A cross-sectional view of the sandwich structure of PU-1A/AgNW/PU-1A in Fig. 5c shows that the nanowires were perfectly embedded at the centre of the sample. We could not find any visible interfaces among the constituent materials. It was impossible to separate the two polymer layers by any physical means. This perfect unity was achieved by the reversible DA reaction designed in this study.

**Figure 6:**
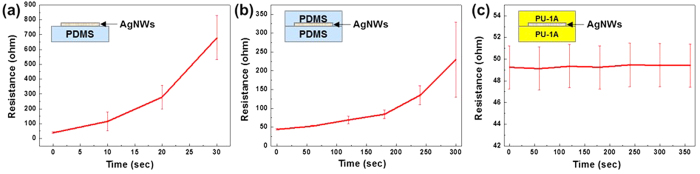
Resistances of various AgNW electrodes measured after immersion into an Ag etchant: (**a**) AgNW/184 PDMS, (**b**) 184 PDMS/AgNW/184 PDMS, and (**c**) PU-1A/AgNW/PU-1A.

To investigate the protective effect of the overlying polymer on the chemical stability of the PU-1A/AgNW/PU-1A structure, the sample was immersed in the Ag etchant. For comparison, AgNW/184 PDMS and 184 PDMS/AgNW/184 PDMS were also tested under the same conditions. As shown in Fig. 6a, the AgNWs directly exposed to the Ag etchant promptly detached from the polymer surface and dissolved, which caused a steep increase in the resistance within a short period. Encapsulation with a 184 PDMS cover layer delayed the increase in the resistance, but the effect became blurred after immersion for 200 s (Fig. 6b). In this case, the liquid permeated through the interfaces of the softly adhered 184 PDMS layers, which allowed the nanowires to come in contact with the liquid. However, the sandwich structure of PU-1A/AgNW/PU-1A was not affected by the immersion, as plotted in Fig. 6c. The test results employing acetone and a commercial stripper are also given in Supplementary Figure S10. The sample was not damaged at all, even with immersion up to 1 h. This means that the AgNWs on PU-1A were perfectly protected by the PU-1A cover layer.

**Figure 7:**
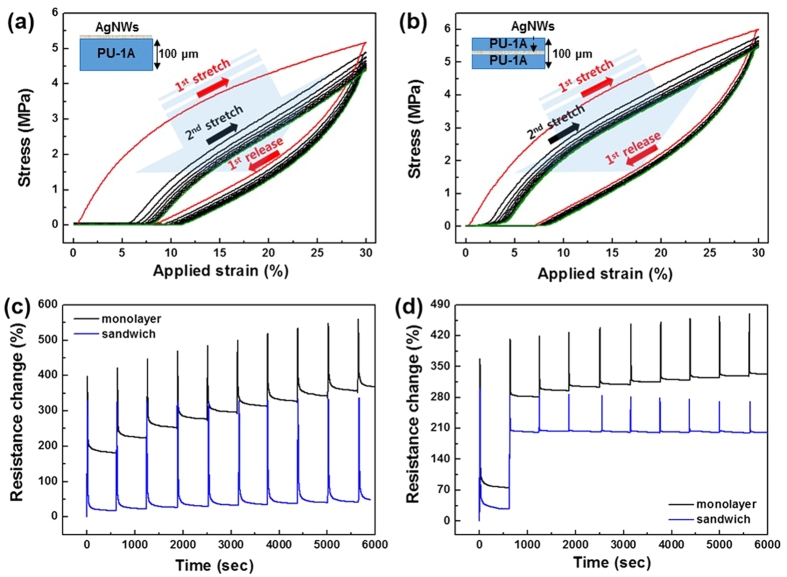
Stress–strain hysteresis curves obtained from cyclic tests (applied strain: 30%) of the (**a**) monolayer (AgNW/PU-1A) sample and (**b**) sandwich (PU-1A/AgNW/PU-1A) sample. (**c**) Resistance change due to repeated stretching and release for an evenly applied strain of 30%. The end of every cycle was followed by a 10 min strain relaxation period. (**d**) Resistance change due to repeated stretching and release. The first cycle was set to stretch the sample by a strain of 30% (displacement rate: 0.5 mm/s) followed by a 10 min strain relaxation period. Then, a 15% strain was repeatedly applied in the following cycles.

Given that the primary goal of developing the PU-1A/AgNW/PU-1A sandwich structure was to obtain highly reliable mechanical performance under continuous large-strain deformation, repeated stretch-and-release tests were carried out for the films fabricated for this study. An automated testing tool was employed to subject the electrode to alternating stretching and releasing repeatedly to induce cyclic fatigue failure. This test used an elongation of 30% and a displacement rate of 0.5 mm/s. First, we investigated the stress–strain behaviour by employing continuous stretch-and-release testing without an interval between cycles. Figure 7a,b show the hysteresis curves for the monolayer and sandwich structures, respectively. Both structures had equal thicknesses of 100 μm. As shown in Fig. 7a, the loading and unloading behaviour of the monolayer was highly nonlinear and showed a large residual strain of approximately 9% after the applied strain of 30% for three main reasons: the high hard segment (**1**) within the polymer, the surface buckling instability mainly caused by the asymmetric structural configuration, and the damaged AgNWs, as shown in Fig. 3. The residual strain was fully recovered within 1 h after the test. However, a rapid response to mechanical loading and unloading is one of the most important factors if the structure is to be employed in the fabrication of strain-based sensor applications. This implies that the residual strain needs to be lowered if possible. Beginning with the second cycle, the hysteresis curve continuously converged, and reversibility was observed after five cycles. Interestingly, Fig. 7b shows that the successful encapsulation by the PU-1 overcoat suppressed the residual strain to 7% after the first cycle. Furthermore, recovery was rather quick, and the stress began to increase at only 2% strain at the beginning of the second cycle. The hysteresis curves overlapped more quickly, and reversibility was certainly observed within a few cycles. Considering that the constituents of the polymer did not differ from the case with the monolayer, this was caused by structural differences. The designed sandwich structure resulted in a more uniform distribution of strain (or stress), a higher resistance to surface buckling, and less damage to the embedded nanowires.

This was also observed in the resistance of the electrodes measured during the stretch-and-release testing. Figure 7c shows the resistance change with repeated testing for an evenly applied strain of 30%. At the end of every cycle, the samples were left unloaded for 10 min to provide a strain relaxation period for the electrodes to recover. For both structures, the resistance abruptly increased with loading and decreased with unloading, which indicates that they can serve as good strain sensors. The highest and lowest peaks continuously increased with continued cycles for the monolayer, whereas they were largely suppressed with the sandwich structure. If the later one is used as a strain sensor, it could perform well under dynamic loads with a high linearity and low hysteresis, even at a large strain (30%). Encouraged by this, we calculated the GF, which is defined as the ratio of the relative change in the electrical resistance to the mechanical strain. The sensor exhibited strong a piezoresistivity with a GF greater than 11. A remaining issue which needed to be addressed was the slow strain relaxation during each resting stage. In an effort to resolve this, we reduced the loading strain to 15% from the beginning of the second cycle, as shown in Fig. 7d. For the monolayer, the hysteresis, irreversibility, and strain relaxation were still observed but reduced. However, they were completely removed from the second loading cycle for the sandwich structure.

**Figure 8:**
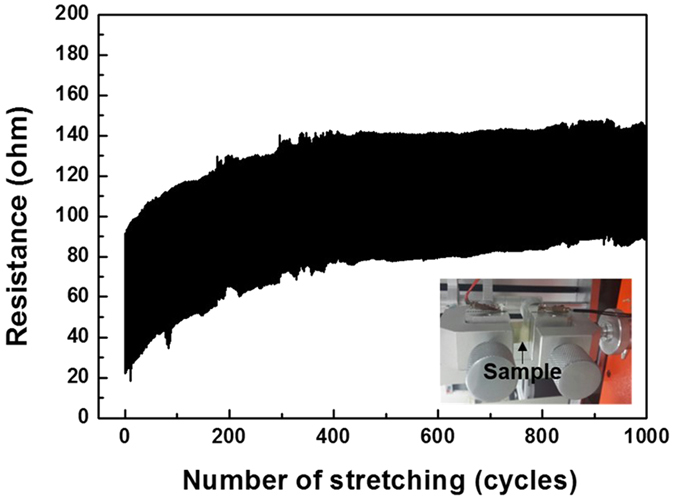
Resistance measured with repeated cycles of stretch-and-release testing of the sandwich structure (strain: 50%).

To evaluate the long-term reliability of the sandwich structure in a stretching environment, a cyclic test of up to 1000 cycles was conducted without a resting period, as shown in Fig. 8. A tensile strain of 50% was used, and stretching occurred at a cycle rate of 0.1 Hz. The resistance was measured 10 times per cycle. The overall resistance of the AgNW network moderately increased at first but stabilised after approximately 400 cycles. This high mechanical stability and reversibility originate from the unique symmetric structure of the PU-1A/AgNW/PU-1A electrode; fully embedding the nanowire electrode at the centre of the elastomeric polymer creates an enlarged interfacial area between the electrode and the polymer. By employing the reversible DA reaction, the two layers of the stretchable elastomer can be successfully united, which also contributes to the high mechanical stability. Because the application of DA adducts into polymers is simple and easily scalable, we believe that our suggested method has the potential to reliably produce stretchable and transparent electrodes. The electrode fabrication scheme is a good example of a solution to major challenges for metal-nanowire-based transparent stretchable electrodes, and the demonstrated approach should serve as a guideline for the fabrication of stretchable electronic devices.

## Conclusion

By crosslinking highly transparent and stretchable silicone with DA adducts, we successfully fabricated a sandwich-structured transparent electrode which is chemically and mechanically stable. Because the DA reaction is reversible, the crosslinked film can react with the resin overcoat to bond with the polymer. Thanks to the perfect joining of the two layers, AgNWs can be fully embedded at the centre of the polymer and sealed from any surrounding liquid. Furthermore, the electrode is mechanically stable, even when stretched by tensile strains greater than 50%. Because the developed electrode varies sensitively and reversibly with the resistance, it can be used as a transparent strain sensor. It performed well under dynamic loads with a high linearity and low hysteresis, even at large strains with a GF greater than 11. The sensor successfully withstood a cyclic stretching test of 50% strain for up to 1000 cycles with a high mechanical stability and reversibility. Considering that our developed concept is largely scalable and can be applied to many other polymers, it has the potential to be a universal method which can be employed to fabricate many other functional structures.

## Methods

### Materials

Silicone polyol (DMS-C15) was purchased from Gelest, USA. IPDI, DMF, glycerol 1,2-carbonate, and furfurylamine were purchased from Tokyo Chemical Industry, Japan. DMF-d7, dibutyltin dilaurate, and BMI were purchased from Sigma-Aldrich, USA. All chemicals were used as received without purification. An AgNW solution dispersed in IPA was purchased from Dittotechnology Ltd., Korea. The average diameter and length of the nanowires were 35 nm and 20 μm, respectively.

### Synthesis of Diol 1

Diol 1 was synthesised following the reported method^15^. A mixture of furfurylamine (4.9 g, 50.8 mmol) and glycerol 1,2-carbonate (6.0 g, 50.8 mmol) was stirred at 60 °C for 3 h. This mixture was used for the next step without purification.

### Synthesis of PU-1A

A flask was charged with IPDI (0.9 g, 4.0 mmol) and DMF (2.9 g). When the solution was clear, diol 1 (0.43 g, 2.0 mmol), silicone polyol (2.0 g, 2.0 mmol), and dibutyltin dilaurate (cat.) were added to the flask. The mixture was stirred at 60 °C for 2 h to afford a DMF solution of PU-1. This solution was mixed with BMI (0.36 g, 1.0 mmol), stirred at 60 °C for 20 min, spin-coated onto a glass substrate, and kept at 50 °C overnight to afford PU-1A.

### Fabrication of the PU-1A/AgNW/PU-1A Electrode

**Figure 9:**
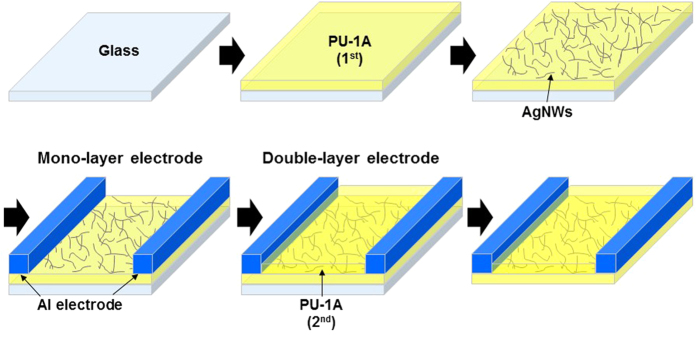
Schematic of the fabrication procedure for the PU-1A/AgNW/PU-1A stretchable electrode.

Figure 9 schematically illustrates the procedure used to fabricate the PU-1A/AgNW/PU-1A transparent electrode. First, glass was cleaned with detergent, deionised water, acetone, and isopropanol. The PU-1 solution mixed with BMI was spin-coated onto the glass and kept at 50 °C overnight. The AgNW dispersion was spin-coated onto the afforded PU-1A and heated in an oven at 120 °C for 10 min to remove any remaining organic solvent from the coating layer. Then, two pieces of Al tape were attached to two sides of the samples, and an overcoat of the PU-1 solution mixed with BMI was applied. The samples were kept at 50 °C overnight and then peeled from the glass.

### Evaluation of the Electrode

IR spectra were obtained by using a Fourier-transform infrared (FTIR) spectrophotometer (IRAffinity-1S, Shimadzu, Japan). Proton nuclear magnetic resonance (NMR) spectra were measured at 400 MHz with an NMR spectrometer (AscendTM 400, Bruker, Germany). An FESEM (JSM6700F, JEOL Ltd., Japan) was used to investigate the microstructure of the AgNW networks. Surface profiles were measured with a laser confocal microscope (VK-9710K, Keyence, Japan). The optical transmission was measured by using an ultraviolet (UV)–visible spectrophotometer (V-560, Jasco, Japan), whereas the sheet resistance *R*_*s*_ was recorded with a non-contact measurement system (EC-80P, Napson Corporation, Japan). An automatic stretch-testing machine (Stretching Tester, Jaeil Optical System, Korea) was used to measure the long-term reliability under repeated cycles of stretching. The electrodes were stretched and released at a rate of 0.5 mm/s under a varying strain to measure the stress–strain behaviour, and the resistance during testing was measured. A long-term cyclic test of up to 1000 cycles was also conducted. This test used a tensile strain of 50% and stretched the electrodes at a cycle rate of 0.1 Hz. The resistance was measured 10 times per cycle. More than 10 samples were fabricated and measured to determine most of the parameters.

## Additional Information

**How to cite this article**: Heo, G. *et al.* Critical Role of Diels–Adler Adducts to Realise Stretchable Transparent Electrodes Based on Silver Nanowires and Silicone Elastomer. *Sci. Rep.* **6**, 25358; doi: 10.1038/srep25358 (2016).

## Supplementary Material

Supplementary Information
