# Supplementary material for: Critical Role of Diels–Adler Adducts to Realise Stretchable Transparent Electrodes Based on Silver Nanowires and Silicone Elastomer
Source: Sci Rep. 2016 May 3;6:25358. doi: 10.1038/srep25358 (PMC4853712; doi:10.1038/srep25358)
Supplement: Supplementary Information [file srep25358-s1.pdf]

Supplementary Information

Critical Role of Diels–Adler Adducts to Realise Stretchable Transparent Electrodes Based on Silver Nanowires and Silicone Elastomer

Gaeun Heo, Kyoung-hee Pyo, Da Hee Lee, Youngmin Kim, and Jong-Woong Kim

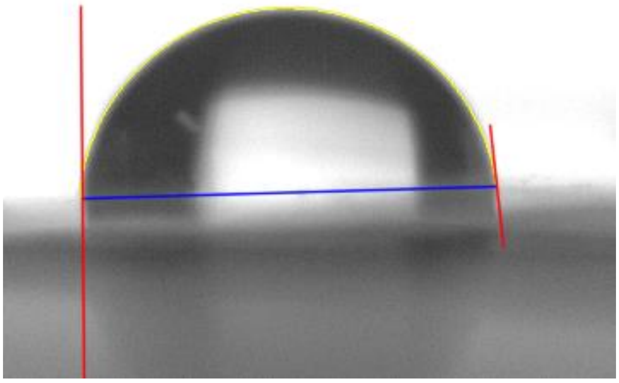

Supplementary Figure S1. The water contact angle of PU-1A. θ = 87.04°.

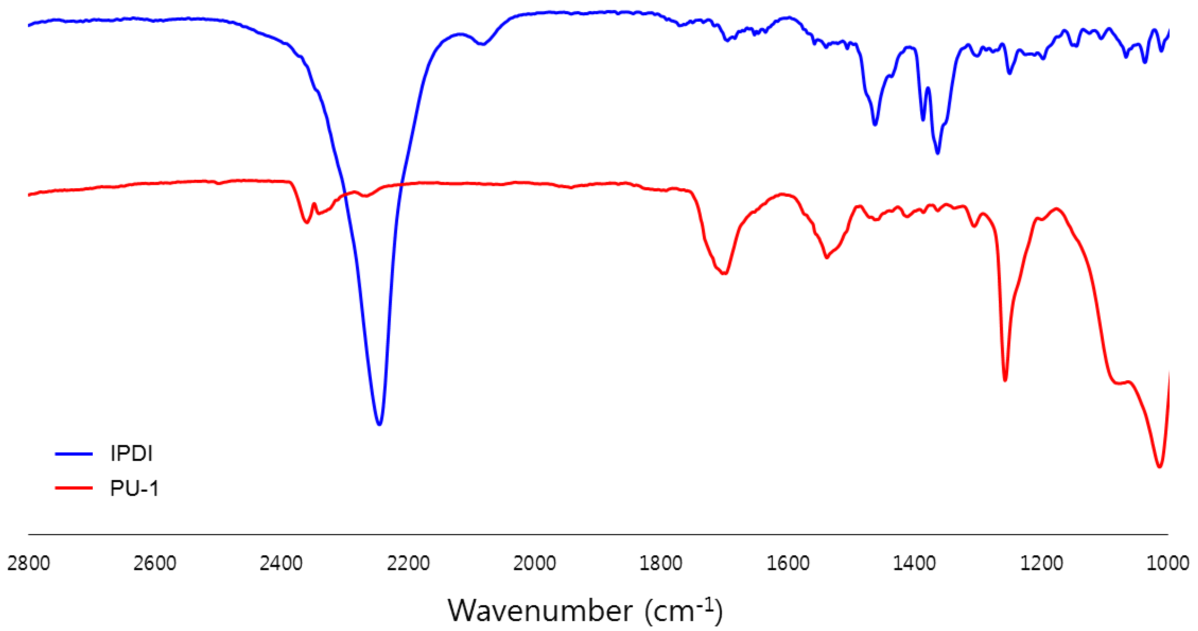

Supplementary Figure S2. FTIR spectra of (blue) IPDI and (red) the PU-1 compound. The absorption intensity at 2260 cm^{-1} corresponding to NCO stretching was reduced in PU-1 compared to that in IPDI.

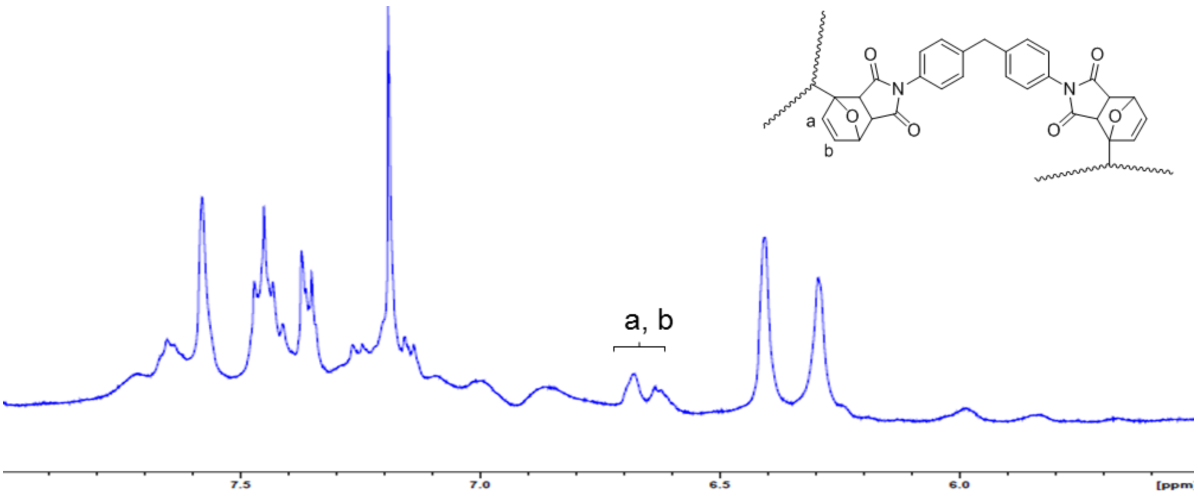

Supplementary Figure S3. ^{1}H-NMR spectrum of the DMF-d_7 solution of PU-1A which was heated at 120 °C for 15 min. PU-1A was not soluble in DMF-d_7 but soluble after being heated owing to the reverse DA reaction which cleaved the DA adducts into furane and maleimide. The remaining DA adduct exhibited peaks at 6.64 ppm and 6.68 ppm. The peaks at 6.29 ppm and 6.41 ppm corresponded to furanyl groups. The peaks at 7.36 ppm and 7.45 ppm corresponded to maleimides.

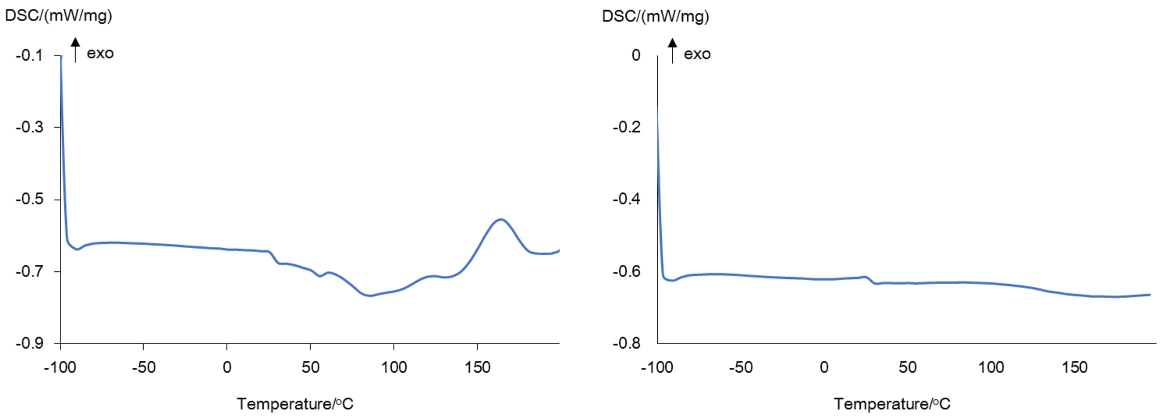

Supplementary Figure S4. DSC curves of PU-1A obtained at the heating rate of 10 ℃/min. The sample was treated sequentially as follows: (left) the sample was heated to 200 ℃, then cooled to -100 ℃, and then (right) heated to 200 ℃.

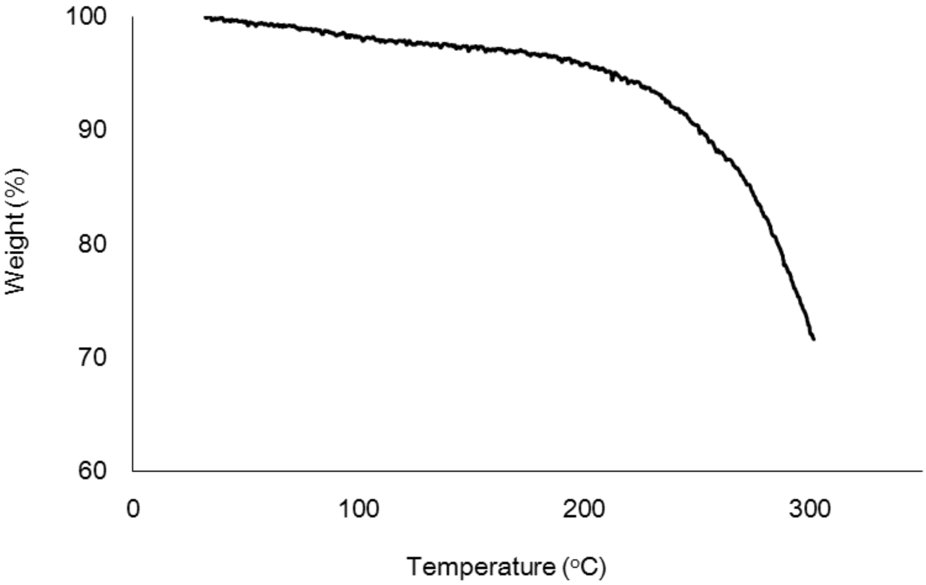

Supplementary Figure S5. TGA data of PU-1A. The weight change was approximately 4% at 200 ℃.

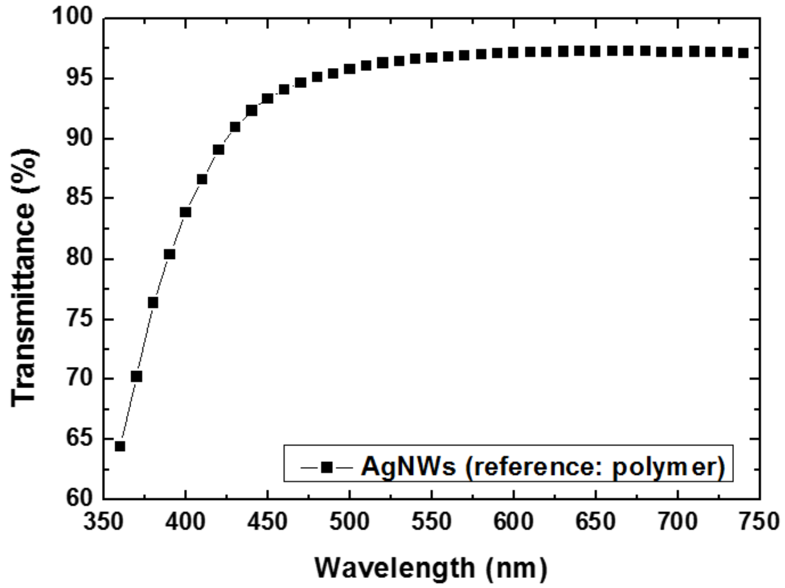

Supplementary Figure S6. Transmittance of the only AgNWs. The reference used for this measurement was the polymer substrate.

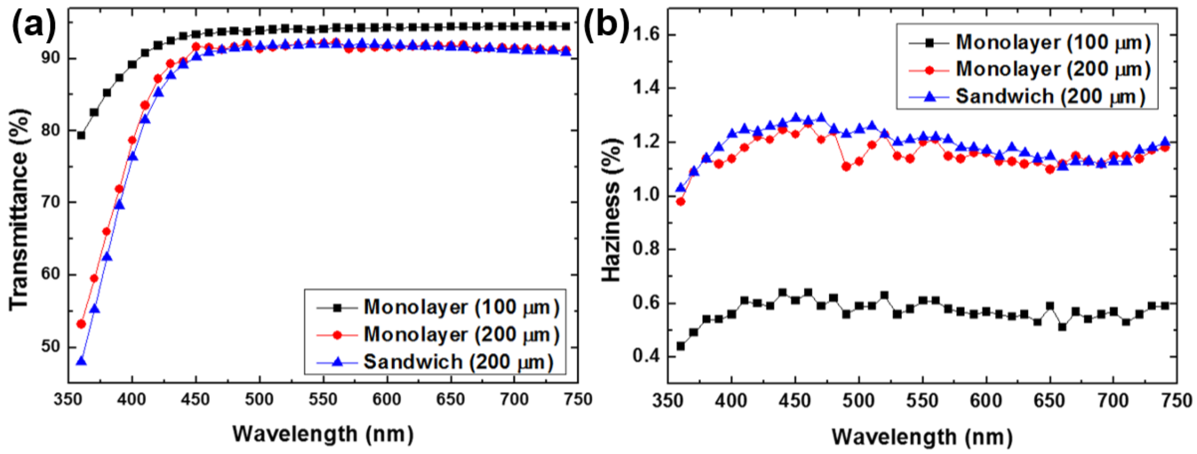

Supplementary Figure S7. (a) Transmittance and (b) haziness of the various structures without employing AgNWs.

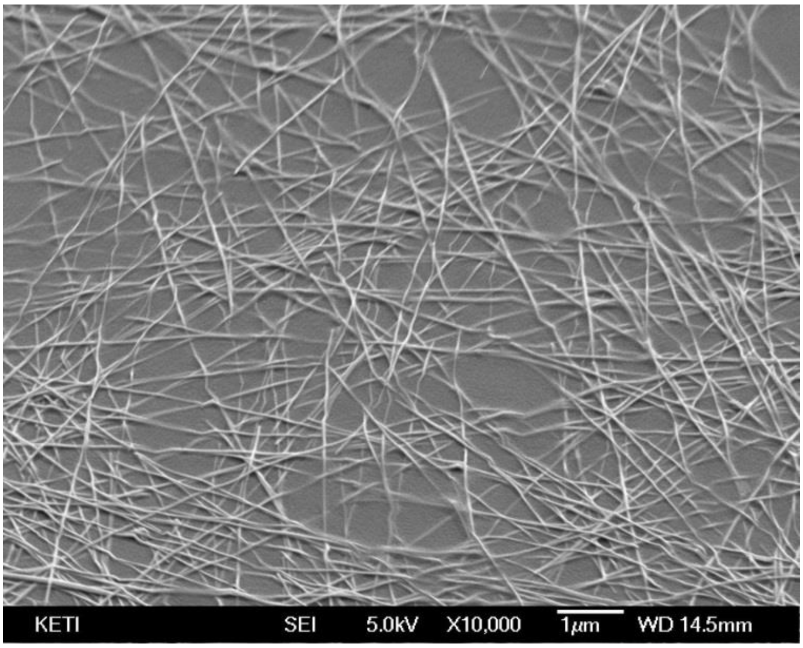

Supplementary Figure S8. SEM micrograph for the as-prepared AgNWs/PU-1A electrode.

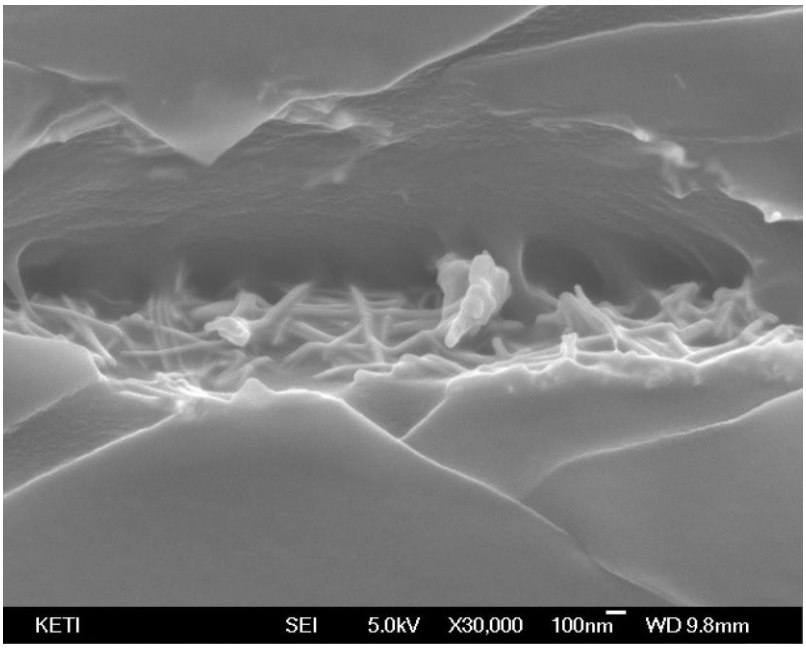

Supplementary Figure S9. Cross-sectional view of the sandwich structure of 184 PDMS/AgNW/184 PDMS after a repeated stretching test with 30% strain for 1000 cycles. The 184 PDMS overcoat starts to peel from the underlying electrode (AgNWs/184 PDMS).

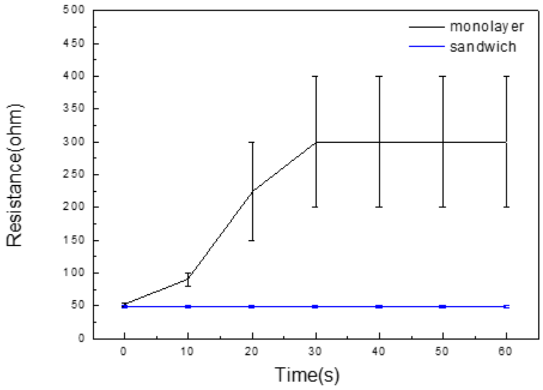

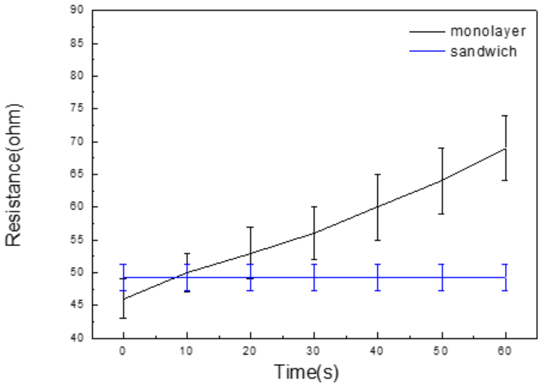

Supplementary Figure S10. Resistance of AgNW electrodes measured after immersion into (a) acetone and (b) a stripper. The sandwich structure was electrically stable, whereas the resistance of the monolayer structure increased during immersion.
